# Inflammation-Accelerated Senescence and the Cardiovascular System: Mechanisms and Perspectives

**DOI:** 10.3390/ijms19123701

**Published:** 2018-11-22

**Authors:** Rita Del Pinto, Claudio Ferri

**Affiliations:** Department of Life, Health and Environmental Sciences, Division of Internal Medicine and Nephrology, University of L’Aquila, 67100 L’Aquila, Italy; claudio.ferri@cc.univaq.it

**Keywords:** cardiovascular disease, aging, molecular mechanisms, inflammation, longevity genes, nutraceuticals, diet

## Abstract

Low-grade chronic inflammation is a common denominator in atherogenesis and related diseases. Solid evidence supports the occurrence of an impairment in the innate and adaptive immune system with senescence, favoring the development of acute and chronic age-related diseases. Cardiovascular (CV) diseases (CVD), in particular, are a leading cause of death even at older ages. Inflammation-associated mechanisms that contribute to CVD development include dysregulated redox and metabolic pathways, genetic modifications, and infections/dysbiosis. In this review, we will recapitulate the determinants and consequences of the immune system dysfunction at older age, with particular focus on the CV system. We will examine the currently available and potential future strategies to counteract accelerated CV aging, i.e., nutraceuticals, probiotics, caloric restriction, physical activity, smoking and alcohol cessation, control of low-grade inflammation sources, senolytic and senescence-modulating drugs, and DNA-targeting drugs.

## 1. Introduction

The pro-inflammatory drive observed with senescence, already defined as “inflamm-aging” [1], and the phenomenon of immunosenescence, which indicates an age-related decline in several immune functions, are multifactorial events of the older age. Growing evidence indicates that these events realize a self-perpetuating condition that favors the development of acute and chronic age-related diseases, spanning from increased susceptibility to infections, to cardiovascular (CV) and neurological diseases. CV diseases (CVD), in particular, are a leading cause of death even at older ages. Although the direction of the association between low-grade chronic inflammation and CVD is yet to be established, the expanding knowledge in the underlying common mechanisms has posed indication to some possible interventions aimed at slowing inflammation-accelerated senescence and the related burden of diseases. In this review, we will recapitulate the determinants and consequences of the immune system dysfunction at older age, with particular focus on the CV system. We will discuss the dysregulated pathways associated with inflammation that contribute to CVD development. Finally, we will examine the available strategies to counteract accelerated CV aging.

## 2. Mechanisms Behind Inflammation-Accelerated Senescence

### 2.1. Possible Triggers to Dysfunctional Immune System at Older Age

**Figure 1:**
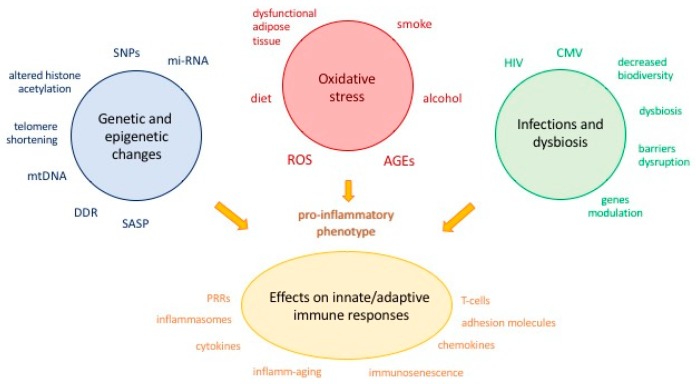
Genetic and environmental factors contributing to changes in the innate and adaptive immune response with age. AGEs: advanced glycation end products; CMV: cytomegalovirus; DDR: DNA damage response; HIV: human immunodeficiency virus; mi-RNA: microRNA; mtDNA: mitochondrial DNA; PRRs: pattern recognition receptors; ROS: reactive oxygen species; SASP: senescence-associated secretory phenotype; SNPs: single nucleotide polymorphisms.

Several genetic and environmental factors contribute to the changes in the innate and adaptive immune response with age (Figure 1).

#### 2.1.1. Genetics

Genetic susceptibility is regarded as a contributor to the inflamm-aging/immunosenescence binomial and its systemic effects. Cytokines—whether pro-inflammatory (i.e., interleukin (IL)-1, IL-6, IL-8, IL-18, IFNα and β, transforming growth factor-β (TGFβ), tumor necrosis factor (TNF) and its soluble receptors) or anti-inflammatory (i.e., IL-1 receptor antagonist (IL-1Ra), IL-4, IL-6, IL-10, IL-11, IL-13, IL-33)—represent crucial players in the immune system network [2]. In fact, they are involved in several scenarios, from endothelial activation to acute phase response, from chemoattraction to anti-inflammatory and antiproliferative pathways. As a consequence, dysfunctional cytokines are among the contributors to impaired immune response with age. Single nucleotide polymorphisms (SNPs) within coding and non-coding regions of their genes are among the best-described mechanisms explaining immune system dysfunction [2,3,4]. Some SNPs are associated with lower, others with higher, CV risk [5,6,7,8,9]. As an example, a study on a functional genetic variant (Asp358Ala) affecting IL6R signaling showed that, for every copy of 358Ala inherited, the risk of coronary heart disease was reduced by 3.4% [6]. Conversely, a meta-analysis on 42 studies including 15,145 cases and 21,496 controls showed that IL-6 gene-174G/C polymorphism was associated with an increased risk of coronary artery disease, especially in Caucasians [10]. Recently, the interaction between cytokines and the renin-angiotensin system (RAS) has also been pointed out as a link between inflammation and the progression of renal and CV diseases [11]. 

Variants in the ILs genes have also been associated with non-CV diseases with an inflammatory component and substantial prevalence at advanced ages, e.g. osteoarthritis (IL-1 region) [12,13], atopy (IL4) [14], rheumatoid arthritis, and asthma (IL6R) [15,16,17]. Cytokines variants have also been found to have an impact on age-related degenerative diseases, as well as on the susceptibility to infections after acute diseases of the older age. In fact, a meta-analysis of 18 case-control studies including 3101 Alzheimer disease cases and 3860 controls showed that genotype CC of IL-6-174G/C was significantly associated with decreased risk of the disease [18]. Finally, a study addressing the effects of the rs4251961 SNP in *IL1RN* on infection risk and outcome in acute stroke patients found that the minor C allele of rs4251961 was independently associated with a decreased risk of non-respiratory infections and with good long-term outcome after 1 year of follow-up [4].

Recently, another interesting finding on the epigenetic regulation of inflamm-aging has been reported, linking decreased acetylation to overexpression of proinflammatory genes during aging [19]. In particular, genes in human and mouse brains with regulatory functions on inflammation appear to be markedly acetylated at the 27th lysine residue of the histone H3 protein (H3K27ac) throughout the genes’ bodies, a property which progressively reduces with aging. This finding indicates that hyperacetylation suppresses overexpression of inflamm-aging genes, suggesting the reversibility of the inflamm-aging process through epigenetic interventions (i.e., histone deacetylase inhibitors) [19]. 

An additional mechanism linking DNA damage to inflamm-aging is telomere shortening [20]. This phenomenon has been shown to contribute to a persistent DNA damage response (DDR) during replicative senescence, which in turn promotes the acquisition of a proinflammatory secretory phenotype at the local and the systemic level. Potential mediators in such association include the micro-RNAs (mi-RNAs), i.e., non-coding RNAs involved in the modulation of gene expression [21]. These molecules have shown ability to promote several proinflammatory pathways, including the nuclear factor kappa-light-chain-enhancer of activated B cells (NF-κB) signaling [21]. An additional circulating mediator of inflamm-aging is mitochondrial DNA (mtDNA): with its ability to bind to pattern recognition receptors (PRRs)—a reminiscence of mitochondrial ancestral bacterial origin—it increases the production of proinflammatory cytokines and activates macrophages [22]. 

#### 2.1.2. Infections and Dysbiosis

Viral infections are one of the triggers to DDR activation [23]. Herpes viruses exploit this mechanism to benefit their replication, thus providing a significant contribution to the accumulation of senescent cells that, in turn, facilitates the development of chronic age-related diseases. As an example, in a cohort of 511 individuals aged ≥65 years who were followed up for 18 years, cytomegalovirus (CMV) infection showed an association with increased mortality, reduced life expectancy by a magnitude of about 3.7 years, and a near doubling of CV deaths [24]. Human immunodeficiency virus (HIV) infection is another contributor to telomeres shortening, and the latter is associated with poorer lung function in HIV-positive patients with chronic obstructive pulmonary disease (COPD), thus suggesting that accelerated aging may be an important driver of lung disease [25]. 

Recent evidence indicates commensal microbial imbalance, i.e., dysbiosis, as another trigger to secondary sustained inflammatory responses related to the development of chronic/autoimmune diseases and cancer [26,27,28]. As an example, inflammatory bowel disease is characterized by a reduction in the number of species within the phylum *Firmicutes* and *Bacteroidetes* and an increase in *Bacillus* spp. and *Enterobacteriacae* [27]. Similarly, dysbiosis and abnormal immune response might be the mechanisms behind periodontal disease, a condition typical of older ages [28]. A key feature of gut microbial changes with age is the reduced biodiversity, with increase in pathobionts and decreased health-promoting bacteria, such as bifidobacteria. There is evidence that aging is associated with a reduction in beneficial commensal microorganisms (i.e., *Coprococcus*, *Faecalibacterium*, *Lactobacillus*, *Firmicutes*) in favour of facultative anaerobes (*Fusobacterium, Staphylococcus*) [29]. This unbalance at the advantage of pathogenic microbial communities disrupts a fine mechanism of mucosal barrier integrity, where fermentation of starches and dietary fibers normally contributes to the production of mucus and lipid metabolites, such as short-chain fatty acids (acetate, propionate, butyrate), which modulate apoptosis and inflammation [29,30]. Diet can be a crucial modifier of the microbiota, with potential impact on the development of disease in predisposed individuals. In particular, high-fat diet was shown to promote tumor progression in the small intestine of genetically susceptible mice, an effect mediated by a shift in the composition of the gut microbiota [31]. 

#### 2.1.3. Oxidative Stress

Cell senescence is also triggered by oxidative stress, consisting of the imbalance between production and clearance of oxidant compounds resulting in macromolecular damage. The hallmark of oxidative stress is the accumulation of reactive-oxygen species (ROS), generated by mitochondrial oxidative respiration and by cellular response to xenobiotics, inflammation, and infections [32,33]. Oxidative stress is implicated in various age-related diseases, such as atherosclerosis, diabetes, cancer, and neurodegeneration, and there is a wealth of evidence indicating its role in shortening life span [34]. In fact, early studies on human fibroblasts revealed that cells grown in the presence of high oxygen concentrations displayed an accelerated rate of telomere shortening, while those grown in low oxygen tension exhibit a prolonged life span [35]. Links between redox unbalance and longevity have been further studied in progressively more complex organisms (*C. elegans*, *Drosophila*, and mice), leading to the identification of many mutated genes involved in regulating energy use [34]. Enhanced activity of antioxidant enzymes (i.e., due to overexpression of glutathione reductase or catalases) appears to be related to increased longevity [34]. Similarly, the splice variant of the adaptor protein Src homology and collagen (Shc) p66^shc^ results in selective resistance to oxidative stress and extended life span, an effect probably mediated by modulation of apoptosis [36]. Interestingly, insulin appears to induce a similar response [37]. Production of angiotensin-II in the vascular wall is another potent mediator of oxidative stress, prompting premature vascular senescence. Interestingly, vitamin D signaling appears to attenuate local production of free radicals, with benefits on vascular health [38]. Periodontal disease and related complications, which are also typical of older age, are another potential source of ROS and chronic, low-grade immune system activation in the elderly [39]. 

Mitochondrial dysfunction, impaired immune function, and chronic, low-grade inflammation are also common features to obesity, and oxidative stress appears to play a central role in this vicious circle. Adipose tissue dysfunction with age can be either the source of oxidative stress and the consequence of low-grade inflammation: in fact, proinflammatory cytokines are able to inhibit adipocyte differentiation, leading to increased ectopic adiposity and elevated serum free fatty acids [40]. In turn, the latter promote a pro-inflammatory state that translates into atherosclerosis and increased CV risk [40]. Growing evidence supports a role for caloric restriction as a measure to break this chain of events, in the hypothesis that it decreases oxidative stress. According to this, limiting food intake was associated to extended life span in a wide range of species and to slower progression of a variety of age-associated diseases [41]. Physical activity is another measure that can reduce systemic inflammation through the modulation of dysregulated chemokine/adipokine secretory patterns and changes in immune cells distribution and phenotype [42]. Exercise can also induce an anti-oxidative microenvironment that limits the propagation of inflammation within the adipose tissue [43]. Similarly, inhibition of lipolysis by sympathetic denervation or through a treatment with a lipase inhibitor appears to significantly decrease ROS-mediated inflammation at the level of adipose tissue [44]. 

### 2.2. Effects on Innate and Adaptive Immunity 

According to the current understanding of the mechanisms behind immune system impairment with senescence, both the innate and adaptive components of immunity are dysfunctional at older age. Defective toll-like receptors (TLRs) responses are one of the crucial features in this scenario, although other innate immune PRRs, such as NOD-like receptor protein 3 (NLRP3) inflammasome activation and the cytoplasmic retinoic acid-inducible gene I (RIG-I)-like receptors, may play an important role [45,46]. TLRs activation normally results in the activation of NF-κB-dependent pathways and the upregulation of interferon-dependent genes, shaping subsequent adaptive T and B cell immune responses. With aging, the expression of TLRs is reduced in several immune cells, with consequent decreased cytokines production, increased susceptibility to viral infections, and also lower response to vaccinations [47]. However, TLR4 appears to be upregulated in aged vascular smooth muscle cells (VSMC), together with chemokines (e.g., C–C Motif Chemokine Ligand 2, CCL2) and adhesion molecules (e.g., Intercellular Adhesion Molecule 1, ICAM1), all involved in the atherosclerotic process [48]. Inflammasome hyperactivation was described in the peripheral blood of older human donors with hypertension, as a potential contributing mechanism to age-associated inflammation and hypertension [49]. The activation of the inflammasome is also observed in cardiac fibroblasts in the early injuries occurring after myocardial ischemia and reperfusion injury, and appears to be dependent on ROS production [50]. 

Age-related changes in the adaptive immune system, i.e., the cellular and the humoral immune responses, include a decrease in naïve T cells, linked to thymic involution, and an increase in highly differentiated CD28- memory T cells, particularly CD8^+^ T cells [51]. CD28-CD8^+^ T cells are characterized by decreased proliferative capacity, shortened telomeres, increased susceptibility to infections, and a weakened immune response to vaccinations. Growing evidence suggests that altered transcription and epigenetic regulation are mechanisms behind T cell senescence [52,53]. These mechanisms include altered miRNA-mediated regulation, changes in histone acetylation, methylation, phosphorylation, or ubiquitination, and decreased DNA methylation, with consequent chromatin structure instability [51]. T-cell receptors sensitivity and signaling are compromised after these changes.

A contribution to the propagation of stress response and pro-inflammatory drive with age comes from a typical feature of senescent cells, i.e., their shift towards a secretory phenotype with paracrine function. Known as senescence-associated secretory phenotype (SASP), it consists in the production of proinflammatory chemokines, cytokines, and extracellular matrix (ECM) proteases, which can amplify the immune activation and contribute to tissue damage [54]. The SASP reflects senescent cells metabolic activity, mirroring the upregulation of the above-mentioned genes and molecular pathways, and represents a potential therapeutic target against accelerated aging. 

## 3. CV Consequences of Inflamm-Aging/Immunosenescence 

**Figure 2:**
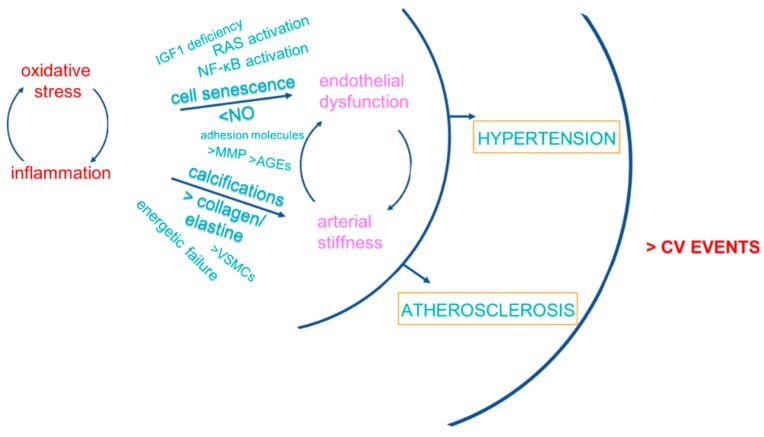
Functional and anatomical CV consequences of inflamm-aging/immunosenescence. AGEs: advanced glycation end products; IGF1: insulin-like growth factor 1; MMP: matrix metalloproteinases; NF-κB: nuclear factor kappa-light-chain-enhancer of activated B cells; NO: nitric oxide; RAS: renin-angiotensin system; VSMCs: vascular smooth muscle cells.

Functional and anatomical CV consequences of inflamm-aging/immunosenescence involve endothelial dysfunction and arterial stiffness, the principal mediators of vascular damage that translates into hypertension and atherosclerosis, leading contributors to CVD (Figure 2).

Endothelial dysfunction is an early marker of vascular aging, preluding the development of hypertension, atherosclerosis of the large vessels, and systemic microvasculopathy [55,56]. Endothelium-derived nitric oxide (NO) acts on VSMCs, regulating arterial resistance and tissue perfusion, but also on the endothelium itself and the immune cells as a modulator of metabolism, signaling, and survival [57]. With aging, oxidative and nitrative stress, as well as disruption of basic metabolic pathways, contribute to endothelial dysfunction [57]. In particular, decreased enzymatic clearance of ROS, i.e., through the inactivation of superoxide dismutase (SOD) or the nuclear factor erythroid 2-related factor 2 (NRF2) pathways, and the decline in glutathione content contribute to endothelial oxidative damage [58,59]. Upregulation of reduced nicotinamide adenine dinucleotide phosphate (NADPH)-oxidase, following pro-inflammatory stimuli or the RAS activation, is in part responsible for oxidative stress at the coronary level [60]. Deficiency of endothelial NO synthase (eNOS) is associated with premature cardiac aging and increased mortality [61]. Senescence-related dysregulation of vascular-specific miRNAs (angio-miRNAs) also shown some relationship with NO synthesis [62]. The activation of NF-κB following low-grade chronic inflammation is another contributor to endothelial senescence, mediated by cytokines and chemokines [63]. In addition, insulin-like growth factor-1 (IGF1) deficiency following age-related decline in growth hormone promotes endothelial dysfunction, microvascular rarefaction and atherosclerosis through redox unbalance and inflammation. There is evidence indicating that IGF-1 reduces atherosclerosis burden and improves features of atherosclerotic plaque stability in animal models [64]. Consistent with these findings, some studies found that low IGF-1 is a predictor of ischemic heart disease and mortality [65,66]. In a population of elderly men in the Netherlands, individuals with the highest IGF-1 bioactivity survived significantly longer, independent of high inflammatory risk profile or a medical history of CV disease [67]. Low free IGF-1 was also associated with increased risk of carotid plaque and coronary artery disease [68]. Taken together, these data indicate that an increase in bioactive IGF-1 is associated with lower atherosclerosis risk and decreased CV mortality. Also, functional failure in the bioenergetic pathway of sirtuins, especially SIRT1, appears to be linked to age-related impaired angiogenesis [57]. This condition has been indicated as a contributor to senescence-related reduction of microvascular density, impaired adaptation to hypoxia, decreased myocardial perfusion, and worsened ischemic tissue damage, at least in part explaining the poorer prognosis of heart failure, myocardial infarction, stroke, and peripheral artery disease at older age [57]. Several pro-angiogenic factors are impaired with aging, including the vascular endothelial and the platelet-derived growth factors (VEGF, PDGF). In the brain, disruption of the endothelium-derived neuropeptide pituitary adenylate cyclase-activating polypeptide (PACAP), a molecule with critical anti-aging effects [69], is associated with impaired angiogenesis [70]. Age-related pericytes loss has been also described as another potential mechanism of impaired microcirculatory network, especially at the level of kidney and brain [57].

Activation of VSMCs following inflammatory stimuli determines their phenotypic transition from the contractile to the synthetic phenotype, which allows their migration from the vascular media to the intima and increases their capacity to generate ECM proteins, with consequent arterial wall thickening [71]. Inflammation-stimulated VSMC can also transdifferentiate into an osteoblastic phenotype, enabling mineralization and calcium deposition in the arterial media, while the activation of matrix metalloproteinases (MMPs) by pro-flogistic mediators determines degradation of elastin and collagen of the vessel wall [72]. All these mechanisms contribute to the phenomenon of arterial stiffness.

## 4. Strategies against Accelerated CV Aging 

**Figure 3:**
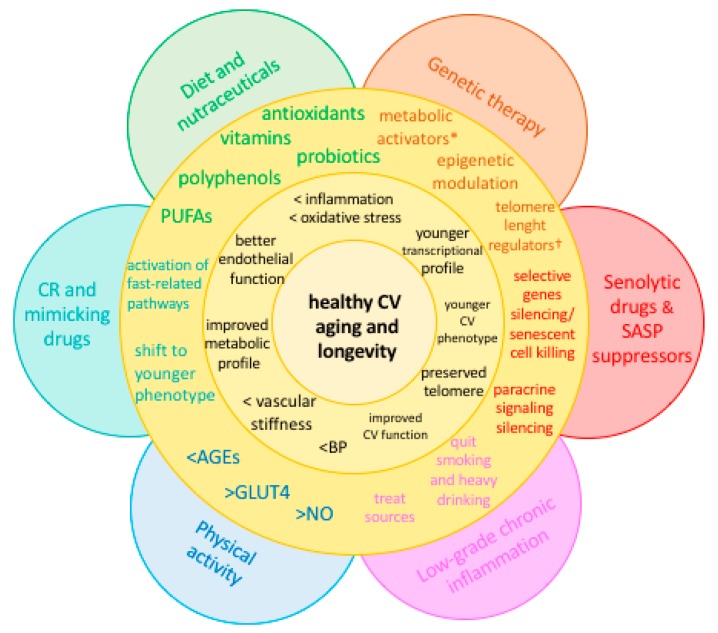
Current and future strategies to preserve the flower of youth. * Sirtuins and mitochondria activators; † telomerase activators. AGEs: advanced glycation end products; BP: blood pressure; CR: caloric restriction; CV: cardiovascular; GLUT4: glucose transporter type 4; NO: nitric oxide; PUFAs: polyunsaturated fatty acids; SASP: senescence-associated secretory phenotype.

Based on the above-mentioned mechanisms of CV aging, several approaches have been investigated to counteract accelerated senescence at the molecular level. Both pharmacological and lifestyle measures showed promising results. Interestingly, while previous data demonstrated substantial benefit over CVD and cancer of traditional pharmacological agents with anti-inflammatory properties (non-steroidal anti-inflammatory drugs, namely aspirin, but also naproxen and colchicine) [30], recent analyses questioned such results, indicating higher all-cause mortality (primarily cancer-related), and no benefit over disability-free survival, among healthy older adults receiving daily aspirin [73,74]. In this setting, alternative treatments or non-pharmacological intervention targeting specific pathways with demonstrated impairment with age become particularly appealing (Figure 3). 

### 4.1. Diet and Nutraceuticals

Evidence from observational studies and intervention trials supports a role for nutraceuticals and specific dietary patterns against accelerated CV aging [75], although more research is needed to clarify the exact underlying mechanisms and to establish dose–response relationships for systematic clinical applications [76]. It appears that the benefits related to the intake of food rich in polyphenols and antioxidants, like whole grains, vegetables, fruits, nuts, fish, and long-chain omega-3 polyunsaturated fatty acids (PUFAs) (i.e., the Mediterranean diet), as well as probiotics, largely depend on their effect on inflammation [75]. Consistent with this, healthy eating habits are associated with lower levels of circulating inflammatory markers, including C-reactive protein (CRP) and several cytokines (IL-6, TNF, IL-18, ICAM-1, vascular cell adhesion protein 1 [VCAM-1], E-selectin) [75,77], and with increased availability of anti-inflammatory mediators (e.g., IL-10, TGF-beta) [78]. Increased intake of PUFAs results in larger incorporation of eicosapentaenoic acid and docosahexaenoic acid into human inflammatory cells phospholipids, partly at the expense of arachidonic acid, resulting in less substrate available for synthesis of the classic inflammatory eicosanoids [79]. Among healthy overweight subjects on a weight-reduction program, and receiving vitamin D (83 μg/d) or placebo for 12 months, a borderline significant reduction in TNF-α concentration (−10% in the treatment group versus −3% in the placebo group) was recorded [80], although this finding was not replicated in subsequent studies [75]. Vitamin E administered together with fish oil for three months was able to blunt the inflammatory response of stimulated blood mononuclear cells in 40 healthy subjects aged >65 years [81]. Regular use of *Lactobacillus delbrueckii* subsp. *bulgaricus 8481* over 6 months among individuals aged >65 years was related to increased number of CD31^+^T-cells, decreased number of CD8^+^CD28-T-cells, and lower rate of CMV reactivation [82]. A decrease in serum IL-8 and an increase in beta-defensin 2 levels were also observed. Other supplementation studies in elderly subjects showed that probiotics (*L. rhamnosus HN001*, *B. lactis HN019, B. lactis Bi-07*, *L. acidophilus NCFM*, *L. casei Shirota*) improved natural killer cells’ cytotoxicity against tumor cells and the macrophage/neutrophil phagocytic activity against *E. coli*. Taken together, these data indicate that probiotic consumption could modulate some pathways of immunosenescence related to innate and adaptive immunity [75]. Additional benefits of the Mediterranean diet include telomere preservation and improved glycolipid profile. In particular, flavanols activate specific NAD-dependent deacetylases, called sirtuins, which are involved in DNA repair and stability, resistance to oxidative stress, mitochondrial energetic pathways, and metabolic dynamics, and appear particularly important for cardiac homeostasis [83]. Resveratrol, a natural SIRT1 activator from grapes, tea, soy, and chocolate [84,85], is thought to prevent the aging-related decline in heart function and neuron loss through anti-inflammatory mechanisms involving a reduction in NF-κB subunit RelA/p65 acetylation [86]. Resveratrol also attenuates the phosphorylation of the mammalian target of rapamycin (mTOR) and S6 ribosomal protein (S6RP), involved in glucose homeostasis [87].

Additional mechanisms for a benefit of nutrients on CV system include the modulation of angiotensin-II signals, with reduced ROS production, mediated by vitamin D [38], and the correction of hyperhomocysteinemia (a new risk factor for CVD and cognitive decline) following vitamin B supplementation [76]. In addition, cinnamon polyphenols have been reported to reverse Tau protein aggregation and dissolve Tau filaments in vitro, and to counteract the increase of amyloid precursor protein in mice brain [88,89]. In a group of 90 cognitively intact older adults, daily consumption of cocoa flavanols for 8 weeks improved cognitive performance, arterial blood pressure, high- to low-density lipoprotein (HDL to LDL)-cholesterol ratio, lipid peroxidation, and insulin resistance. The underlying mechanism likely exploits the positive effect of flavanols on endothelial function, with benefits on insulin sensitivity [90]. In addition, growing evidence suggests that some dietary flavonoids, i.e., epicatechin and epigallocatechin, might delay the onset and/or progression of Alzheimer disease through decreased βγ-secretase activity and Aβ production. The underlying molecular mechanism appears to be mediated by the inhibition of the β-site amyloid precursor protein cleaving enzyme 1 (BACE1) [91]. Neuronal activity during cognitive performance appears to be tightly related to increases in local blood flow, a process known as cerebrovascular coupling. There is increasing evidence suggesting that this effect is also mediated by NO from NOS-containing neurons adjacent to cerebral microvessels [92]. 

### 4.2. Caloric Restriction and Mimicking Drugs 

Caloric restriction (CR) is regarded as an efficient anti-aging approach. The effect of CR on primates was first examined about three decades ago, showing a positive association of CR with survival, with a 2.6-fold increased risk of death in control animals compared to restricted [93]. A recent review incorporating data from subsequent studies confirms this first observation [94]. Accumulating evidence from observational and randomized clinical trials indicates that CR is associated with increased life span also in humans [95]. The underlying mechanism involves a positive effect on multiple metabolic and hormonal factors implicated in the pathogenesis of age-related chronic diseases with the greatest burden of disability and mortality, i.e., CVD, type 2 diabetes, and cancer. In particular, a decrease in total body and intra-abdominal fat, an improvement in insulin sensitivity and glycosylated hemoglobin, a decrease in triglycerides and arterial blood pressure, as well as compositional changes in LDL-cholesterol towards a reduced atherogenic potential, were all common features to CR regimens [96]. A decline in markers of oxidative stress and an improvement in T-cell function (i.e., delayed type hypersensitivity response and proliferative response to specific mitogens) and prostaglandin E2 production, were also observed in restricted diets. Low-glycemic CR showed an association with reduced CRP. As clinical consequences of these cardiometabolic changes, the intima-media thickness of the common carotid arteries was significantly lower after CR compared to usual Western diets, and better echocardiographic markers of left ventricular diastolic function, together with an improvement in the autonomic function in terms of heart rate variability, were reported [97,98]. At the molecular level, several metabolic pathways involved in the accumulation of molecular damage, i.e., SOD, or in the adaptation to CR, i.e., adenosine monophosphate-activated protein kinase/SIRT1 (AMPK/SIRT1), phosphatidylinositol-3-kinase/protein kinase B/mTOR (PI3K/AKT/mTOR), and IGF-1/insulin pathways, underwent changes towards a younger transcription profile promoting health and longevity [99]. In particular, sirtuins are activated, while mTOR is deactivated, during fasting conditions, with consequent fine regulation of autophagy. Modulation of these pathways that also exploits a genetic approach appears to be a particularly promising anti-aging strategy.

### 4.3. Senolytic Drugs and SASP Suppressors

A new class of drugs, termed senolytics, is emerging as a potential anti-aging resource [100]. The rationale for their development is in the increased expression of pro-survival networks in senescent cells, conferring them resistance to apoptosis. Key nodes of this network include ephrins (ephrin B1 or 3, EFNB1 or 3), PI3Kδ, p21, B-cell lymphoma-extra-large (BCL-xL), and plasminogen-activated inhibitor-2 (PAI-2). Silencing these genes using short interfering RNAs (siRNAs) determined the selective killing of senescent cells, without affecting differentiated cells [100]. Drugs successfully targeting these same factors both in vitro and in vivo include dasatinib, quercetin, and navitoclax. The first is an inhibitor of multiple tyrosine kinases and an apoptosis inducer, with preferential killing of senescent human preadipocytes, while quercetin is a natural flavanol with inhibitory properties on PI3K, other kinases, and serpines. In vitro studies showed that dasatinib induced effective clearance of senescent human fat cell progenitors, while quercetin was more effective against senescent human endothelial cells and bone marrow-derived murine mesenchymal stem cells (BM-MSCs); the combination of the two effectively eliminated senescent mouse embryonic fibroblasts (MEFs) [100]. In vivo, this combination reduced senescent cell burden in chronologically aged, radiation-exposed mice [100]. Treating 24-month-old mice with the same combination improved cardiac ejection fraction, endothelium-dependent relaxation, smooth muscle vascular reactivity to NO, and smooth muscle contractile function 5 days after a single dose [100]. The same single administration determined a persistent improvement in treadmill exercise capacity in mice after single leg radiation exposure, while periodic drug administration extended health span in mice, reducing age-related disease burden [100]. Navitoclax, which targets components of the Bcl pathway, was found to reduce viability of senescent human umbilical vein epithelial cells (HUVECs) and lung fibroblasts, but not human primary preadipocytes [101].

Other potential anti-aging drugs include rapamycin, metformin, and Janus kinase 1/2 (JAK1/2) inhibitors, which act as SASP suppressors, thus decreasing the pro-inflammatory drive observed with senescence [102,103]. In addition to this mechanism, JAK1/2 inhibitors (i.e., ruxolitinib) appear to exert potential effects on telomere shortening [104]. All these molecules alleviate metabolic impairments involved in inflamm-aging, including insulin-resistance and adipose tissue dysfunction. Metformin and rapamycin reduce CV risk and cognitive impairment and increase lifespan, while ruxolitinib and rapamycin reduce frailty and immune/stem cells dysfunction [102]. 

Another potential player against CV ageing is IGF-1. IGF-1 deficiency is associated with decreased lifespan and increased incidence of fatal atherosclerotic CV events [105,106], and IGF-1 supplementation with recombinant human IGF-1 analogs (mecasermin and mecasermin rinfabate) appears to efficiently revert CV remodeling and to improve lipid profile [107]. To date, however, these molecules have not been tested for use as anti-aging drugs.

The potential application of the described measures to human aging deserves further investigations. Future directions of a genetic approach to senescence include metabolic modulation (i.e., through the sirtuins and mTOR pathways), and DNA stability and repair (i.e., by telomere preservation). 

### 4.4. Physical Activity

It is well established that reduced physical activity and low aerobic capacity are strong and independent predictors of CVD morbidity and mortality [108,109]. Data from old mice showed that voluntary wheel running increased NO bioavailability while reducing the accumulation of advanced glycation end products (AGEs), that of markers of protein oxidation (i.e., nitrotyrosine), as well as the vascular expression of major oxidative stress enzymes and inflammatory transcription factors (i.e., NADPH oxidase, NF-κB), with consequent lower levels of serum cytokines [110,111]. Both endurance and resistance exercise exert several benefits on cardiometabolic health through anti-inflammatory, antioxidant, and metabolic mechanisms [111]. Aerobic exercise increases mitochondrial biogenesis [112], with consequent increased calorie burning leading to a decrease in abdominal fat and in body mass index (BMI) [113]. It also increases NO availability, reduced markers of inflammation (e.g., CRP), oxidative stress and intercellular adhesion molecules, upregulates the prostanoid system, and is associated to lower systolic and diastolic blood pressure [113,114]. In addition, it can prevent or reverse age-related coronary/large artery stiffening [111,115]. In terms of glycolipid metabolism, aerobic exercise boosts the expression of the insulin responsive glucose transporter type 4 (GLUT4) [112], with increased insulin-sensitivity and improved glucose tolerance, and mediates an increase in the expression of lipoprotein lipase (LPL) in skeletal muscle, leading to a more favorable serum lipid profile [116]. Resistance exercise is also particularly effective in increasing GLUT4-mediated pathways, leading to improvement in glucose tolerance and insulin sensitivity [117]. A potential effect of physical exercise on cognition has also been proposed, with hypothetical induced nerve cells outgrowth and consequent better communication between brain cells, but data on the relationship between dementia and exercise appear inconclusive [118].

### 4.5. Other Potential Sources of Chronic, Low-Grade Inflammation

Another potential source of chronic, low-grade inflammation in the elderly is represented by periodontal disease, a chronic inflammatory disorder of the tissues surrounding the teeth that becomes more prevalent with age [119]. Similarly, biological complications in implant rehabilitation occurs more frequently at older age [120]. Accumulating evidence supports the existence of systemic effects of periodontal inflammation on CV risk factors and diseases. In particular, history of periodontitis has been associated with incident cerebrovascular disease, coronary heart disease, chronic kidney disease, and mortality [119]. Interestingly, periodontitis is associated with a worse systolic blood pressure profile during antihypertensive therapy by about 2.3–3 mmHg and with higher odds of antihypertensive treatment failure [119]. It appears that the accumulation of AGEs during periodontal disease or peri-implantitis is one of the triggers to the cascade of pro-inflammatory signaling that subsequently activates redox-sensitive transcription factors (i.e., NF-κB) responsible for endothelial cells hyper-permeability, VCAM-1 molecules activation, chemotaxis, and cytokines/interleukins (TNF, IL-1, IL-6) release into the bloodstream [39]. Circulating inflammatory mediators, in turn, elicit endothelial dysfunction, with consequent impaired vasodilation and alterations in the vascular structure [119]. Low-grade bacteremia and endotoxemia, as well as cross-reactivity or molecular mimicry between bacterial- and self-antigens, represent additional mechanisms potentially linking periodontal disease to systemic diseases [119]. Thus, oral hygiene measures have been suggested as an additional strategy to control this potential source of chronic inflammation [119].

Smoking and heavy drinking are additional factors related to premature aging through mechanisms involving DNA methylation and repair, as well as redox unbalance following AGEs accumulation [121,122,123]. Both also represent established CV risk factors [124,125]. Thus, smoke or alcohol quitting represent further measures against accelerated CV aging. 

## 5. Conclusions 

With the great burden posed by CVD in terms of human lives and economic costs even at advanced ages, learning the mechanisms behind healthy and unsuccessful CV aging for targeted prevention and treatment is the challenge for the future generations. At present, it appears that lifestyle measures, including eating habits, physical activity, smoking and drinking cessation, but also the control of potentially correctable sources of low-grade inflammation, are safe and effective preventive strategies from a younger age. Dietary supplements, functional foods, and probiotic formulations have aroused interest as non-pharmacological, yet additive, fitness strategies: in this context, the use of products of tested quality and efficacy is of paramount importance [126]. Future directions against accelerated and unsuccessful CV aging are likely to involve a molecular approach: thus, DNA-targeting drugs acting against the age-related dysregulation of inflammatory, redox, and metabolic pathways are promising strategies to preserve the flower of youth. 

## Abbreviations

CVD	cardiovascular diseases
IL	interleukin
IFN	interferon
TNF	tumor necrosis factor
TGF-β	transforming growth factor-β
SNPs	single nucleotide polymorphisms
DDRs	DNA damage response
mtDNA	mitochondrial DNA
mi-RNA	micro-RNA
si-RNA	short interfering RNAs
CMV	cytomegalovirus
HIV	human immunodeficiency virus
ROS	reactive-oxygen species
NO	nitric oxide
SOD	superoxide dismutase
NF-κB	nuclear factor kappa-light-chain-enhancer of activated B cells
TLRs	toll-like receptors
CR	caloric restriction
AGEs	advanced glycation end products
